# The association of intra-therapeutic heterogeneity of somatostatin receptor expression with morphological treatment response in patients undergoing PRRT with [^177^Lu]-DOTATATE

**DOI:** 10.1371/journal.pone.0216781

**Published:** 2019-05-15

**Authors:** Christoph Wetz, Philipp Genseke, Ivayla Apostolova, Christian Furth, Sammy Ghazzawi, Julian M. M. Rogasch, Imke Schatka, Michael C. Kreissl, Frank Hofheinz, Oliver S. Grosser, Holger Amthauer

**Affiliations:** 1 Department of Radiology and Nuclear Medicine; University Hospital Magdeburg A.ö.R., Otto-von-Guericke University Magdeburg, Magdeburg, Germany; 2 Department of Nuclear Medicine, Charité-Universitätsmedizin Berlin, Berlin, Germany; 3 Department of Nuclear Medicine, University Medical Center Hamburg UKE, Hamburg, Germany; 4 Helmholtz-Zentrum Dresden-Rossendorf, Institute of Radiopharmaceutical Cancer Research, PET Center, Dresden, Germany; Chang Gung Memorial Hospital at Linkou, TAIWAN

## Abstract

**Aim:**

Purpose of this study was to evaluate the association of the spatial heterogeneity (asphericity, ASP) in intra-therapeutic SPECT/ CT imaging of somatostatin receptor (SSR) positive metastatic gastroenteropancreatic neuroendocrine neoplasms (GEP-NEN) for morphological treatment response to peptide receptor radionuclide therapy (PRRT). Secondly, we correlated ASP derived form a pre-therapeutic OctreoScan (ASP_[In]_) and an intra-therapeutic [^177^Lu]-SPECT/CT (ASP_[Lu]_).

**Materials and methods:**

Data from first therapy cycle [^177^Lu-DOTA^0^-Tyr^3^]octreotate ([^177^Lu]-DOTATATE)-PRRT was retrospectively analyzed in 33 patients (m = 20; w = 13; median age, 72 [46–88] years). The evaluation of response to PRRT was performed according to RECIST 1.1 in responding lesions [RL (SD, PR, CR), n = 104] and non-responding lesions [NRL (PD), n = 27]. The association of SSR tumor heterogeneity with morphological response was evaluated by Kruskal-Wallis test and receiver operating characteristic curve (ROC). The optimal threshold for separation (RL vs. NRL) was calculated using the Youden-index. Relationship between pre- and intra-therapeutic ASP was determined with Spearman’s rank correlation coefficient (*ρ*) and Bland-Altman plots.

**Results:**

A total of 131 lesions (liver: n = 59, lymph nodes: n = 48, bone: n = 19, pancreas: n = 5) were analyzed. Lesions with higher ASP values showed a significantly poorer response to PRRT (PD, median: 11.3, IQR: 8.5–15.5; SD, median: 3.4, IQR: 2.1–4.5; PR, median 1.7, IQR: 0.9–2.8; CR, median: 0.5, IQR: 0.0–1.3); Kruskal-Wallis, p<0.001). ROC analyses revealed a significant separation between RL and NRL for ASP after 4 months (AUC 0.85, p<0.001) and after 12 months (AUC 0.94, p<0.001). The optimal threshold for ASP was >5.45% (sensitivity 96% and specificity 82%). The correlation coefficient of pre- and intra-therapeutic ASP revealed ρ = 0.72 (p <0.01). The mean absolute difference between ASP_[In]_ and ASP_[Lu]_ was -0.04 (95% Limits of Agreement, -6.1–6.0).

**Conclusion:**

Pre- and intra-therapeutic ASP shows a strong correlation and might be an useful tool for therapy monitoring.

## Introduction

Neuroendocrine neoplasms of the gastroenteropancreatic system (GEP-NEN) represents a rare and heterogeneous tumor entity. However, the incidence of GEP-NEN increased recently, mainly due to improved functional imaging and device-specific sensitivity[1]. Beyond commonly “cold” agents, such as somatostatin analogs, as a first-line antiproliferative drug, peptide receptor radionuclide therapy (PRRT) with [^177^Lu-DOTA^0^-Tyr^3^]octreotate ([^177^Lu]DOTATATE) has emerged as a highly effective treatment in metastatic well-differentiated GEP-NEN of low and intermediate grade G_1_ and G_2_. [2, 3]. Several retrospective studies could also show the superiority of [^177^Lu]DOTATATE-PRRT in advanced inoperable GEP-NEN compared to other treatment modalities[4–6]. The recently published results of the first prospective randomized study in patients with progressive metastatic midgut NEN, NETTER-1, found a rate of 65.2% progression-free survival at month 20 after PRRT. In particular, the overall survival (OS) has not yet been reached in the study cohort [7]. In contrast, the current published ENETS guidelines recommend the use of PRRT in intestinal (midgut) NEN with distance and/ or loco regional metastases as a second- to third-line therapy after progression under somatostatin-analogues (SSA) irrespective of the abovementioned NETTER-1 results. According to guidelines in pancreatic NEN with advanced locoregional disease PRRT should be even used as a third line therapy after failure of SSA, everolimus and/ or cytotoxic chemotherapy [8].

Meanwhile, application of PRRT can be only repeated to a limited extent, imposed by critical dose exposure in different organs at risk, e.g. in bone marrow (<2Gy) [9, 10] in 70% of patients after treatment with [^177^Lu]DOTATATE and in kidney (<40 Gy) [11]. Histological diversities in metastatic SSR-expression are common findings, and may deliver objective prediction of therapy efficacy [12, 13]. However, not all patients benefit from PRRT. Therefore, better stratification criteria are highly desirable to identify patients with high probability for tumor response.

Until now, diagnosis and staging of NEN are merely based on analysis of tumor spread and morphology in imaging, irrespective of SSR expression and density.

Promising results have been published considering features from the non-invasive SSR-imaging that can be used separately or in combination to characterize more accurately biological behavior of NEN, thus leading to a better understanding of tumor/therapy response. The Krenning score, a well-established qualitative measure [3, 14], the metastases to liver ratio (M/L ratio), a first scanner-independent quantitative surrogate in [^68^Ga-DOTA^0^-Phe^1^-Tyr^3^]octreotide (DOTATOC)-positron emission tomography (PET) combined with computed tomography (CT) [15], as well as the intratumoral SSR-heterogeneity in [^68^Ga]DOTATATE-PET/CT [16] represent the most common methodologies in prediction of response in PRRT. The assessment of asphericity (ASP), characterizing lesion’s spatial SSR-heterogeneity e.g. by [^111^In-DTPA^0^]octreotide ([^111^In]octreotide, OctreoScan) scintigraphy [13], demonstrated an further promising parameter to predict response in PRRT planning [17]. Whereas, translation and verification of this novel image based methodology to intra-therapeutic workflow is missing.

The aim of the present study was to evaluate the predictive capability of tumoral SSR-receptor heterogeneity based on intra-therapeutic imaging with [^177^Lu]DOTATATE. Secondly, we correlated ASP derived form a pre-therapeutic OctreoScan (ASP_[In]_)and an intra-therapeutic [^177^Lu]DOTATATE-SPECT/CT imaging (ASP_[Lu]_).

## Materials and methods

### Patients

In this monocentric setting, data of all consecutive patients (n = 33; f, n = 13; m = 20; median age 72± 9.2 years, range, 46 to 88 years) treated by PRRT using [^177^Lu]DOTATATE were retrospectively included in the period from June 2011 to September 2015. The patients fulfilled the following inclusion criteria: (1) intra-therapeutic SPECT/CT (24 hours p.i.), (2) metastases could be clearly delineated on SPECT imaging (33/37 patients; 89%), (3) GEP-NEN was histologically proven, (4) patients underwent 2–4 cycles PRRT, (5) radiological follow up at least 12 months was available (33/41 patients; 80,5%). The assessment of tumor SSR-positivity was determined by [^111^In]octreotide SPECT/CT or [^68^Ga]DOTATOC-PET/CT prior to therapy. Further analysis was performed in a subset of twenty patients (f, n = 14; m = 6; median age 72± 7.4 years, range, 54 to 87 years) for a lesion-based correlation of ASP from pre-therapeutic [^111^In]octreotide SPECT/CT with intra-therapeutic SPECT/CT.

All patients provided written informed consent on the evaluation of their data, and approval from the institutional ethics committee of the Otto-von-Guericke-University (reference ID: RAD279; vote, 07/16) was obtained.

### ^177^Lutetium—Peptide receptor radionuclide therapy (PRRT)

All patients underwent PRRT in concordance to the Rotterdam protocol and *The joint IAEA*, *EANM and SNMMI practical guidance* [18] as recently described [13]. In brief, patients obtained a ^177^Lutetium-labelled (T_1/2_ = 6.7d) synthetic somatostatin analogue, [DOTA^0^-Tyr^3^]octreotate. The patients were treated by two to four cycles (median, 3.3; IQR 3–4; range 2–4) of radionuclide therapy with a median administered activity of 200 mCi (7.4 GBq) [^177^Lu]DOTATATE per cycle. Follow up including both morphological contrast-enhanced CT (CE-CT) and functional imaging [^111^In]-OctreoScan, n = 12/33, 36%, or alternatively [^68^Ga]DOTATOC-PET/CT, n = 15/33, 64%, was obtained after the second and fourth cycle and 4 and 12 months, respectively. If interim staging indicated progressive disease, no further cycle of PRRT was administered [13].

### SPECT/CT imaging

Intra-therapeutic imaging was performed with a dual head SPECT/CT (Discovery NM/CT670, GE, Haifa, Israel) according to standard protocols. Planar imaging and SPECT with low-dose CT (X-ray tube current = 40 mAs) of thorax and abdomen for morphological correlation was carried out 24 hours p.i.. SPECT data were reconstructed by iterative algorithms with CT-based correction for attenuation as previously described [13]. Imaging data was evaluated with a dedicated workstation (Xeleris-Workstation, GE Healthcare, Waukesha, USA) at standard clinical settings. Diagnostic CE-CT as well as contrast enhanced MRI of the liver was performed according to a standard protocol, published elsewhere [13, 19].

### Image analysis

Intra-therapeutic SPECT data sets were evaluated regarding SSR-heterogeneity in accumulation pattern as previously described [13]. In brief, SPECT data measured 24h p.i. at the first cycle [^177^Lu]-DOTATATE-PRRT was analyzed by a dedicated software (ROVER version 2.1.20, ABX, Radeberg, Germany). The functional tumor volume, a measure of the SSR uptake of tumor tissue, was delineated by a semi-automatic algorithm based on adaptive threshold taking the local background into account [20, 21]. For the resulting volumes of interest (VOI) the ASP was computed using the following equation:

$$ASP=\sqrt[{3}]{H}-1\;with\;H=\frac{1}{36\pi }\frac{S^{3}}{V^{2}}$$

where S and V are the mean surface and volume of the lesions’ functional active part, respectively. The ASP described the deviation of an activity accumulation from spherical geometry. A small ASP (Range 1–5%) represents a more spherical accumulation while increasing ASP represents considerably deviating pattern (e.g. elliptical). A more detailed description has been given in recent studies [13, 22].

For lesion based analysis the heterogeneity of lymph-node-, bone-, liver- as well as pancreatic- metastases were assessed to ensure a systemic and not organ-based approach. According to the response evaluation criteria in solid tumors (RECIST 1.1) only two lesions per organ and not more than five per patient were included. In the case of more than two metastases per organ ASP was derived from two individual lesions with the most representative SSR-uptakes on visual assessment.

The association between ASP and results from RECIST-evaluation was evaluated for three different time points (pre PRRT: mean = 4 weeks, range: 1–9 weeks), 4 months post PRRT, and 12 months post PRRT). In generally, morphological assessment was performed by CE-CT. Alternatively, CE-MRI was used for morphological evaluation (contrast-enhanced, CE-MRI; n = 23/33 70%) if available. Analysis of the CE-CT (CE-MRI) data was performed using dedicated radiologic workstation INFINITT (INFINITT Healthcare Co., Ltd., Seoul, South Korea). In order to permit comparison with CE-CT, generally the short axis of lymph-nodes and long diameters in the transverse plane for liver, pancreatic and bone metastases were drawn. Lesions with a short axis of <15 mm (lymph nodes) and <10 mm (pancreas, liver lesions, bone lesions) were not included into analysis to avoid partial volume effects. The morphological changes in diameter were classified according to the RECIST 1.1 guidelines. Definition of VOIs and calculation of the diameter were performed in consensus by two physicians. The different classes were further cumulated in responding lesions (RL [SD, PR, CR], n = 104) and non-responding lesions (NRL [PD], n = 27). The rationale for this definition was published elsewhere [23].

### Statistical analysis

Data analyses were performed using SPSS 22 (IBM Corp., Armonk, NY, USA). Descriptive values were expressed as median, interquartile range (IQR, 25th percentile–75th percentile), and range (minimum–maximum) and depicted as boxplots. According to histograms and quantile-quantile plots, the distribution of data-sets was assumed non-parametric. The ASP within the context of intra-therapeutic dosimetry was analyzed using the Kruskal-Wallis test and receiver operating characteristic curve (ROC). The optimal threshold for separation of RL and NRL was calculated using the Youden’s index [24].

The cutoff values were assessed separately for prediction response at 4 and 12 months post PRRT. ASP values was binarized using ROC cutoffs and the associated sensitivity and specificity were determined. Statistical significance was assumed at at a *P* value < 0.05 and high significance at *P* value < 0.001. A subanalysis was performed in patients who underwent a pretherapeutic [^111^In]octreotide SPECT/CT. The correlation of ASP_[In]_ and ASP_[Lu]_ was compared by Spearman's rank correlation coefficient rho (*ρ*) and the limits of agreement were determined in a Bland-Altman diagram. Concordance was assumed at a deviation of ≤ 5%.

## Results

Of the 33 patients, 10/33 (30.3%) patients had a GEP-NEN including the primary tumor of the foregut (stomach, pancreas and duodenum); in 13/33 (39.4%) patients, the primary occurred in the midgut (jejunum, ileum, and proximal colon); in 5/33 (15.2%) patients, the tumor origin was localized in the hindgut (the remaining two thirds of colon and rectum). 5/33 (15.2%) patients suffered from cancer of unknown primary (CUP). All NENs were well differentiated, low (G_1_) to intermediate (G_2_) grade. Antigen Ki-67, the proliferation index, ranged from 1–17% (median 4%). Chromogranin A (CgA) levels ranged between 94–12600 μg/l (median 632). All patients showed inoperable, metastasized NEN and had undergone different treatment modalities such as surgical intervention (n = 29/33, 87.9%), somatostatin analogue therapy (n = 31/33, 93.9%) or chemotherapy (n = 6/33, 18.2%). Routinely acquired patient data and characteristics are illustrated in Table 1.

**Table 1:** Patient data and therapy characteristics.

Characteristic		Value
**Primary**		**33 (100%)**
	**GEP-NEN Total (n)**	
	foregut	10 (30.3%)
	midgut	13 (39.4%)
	hindgut	5 (15.15%)
	**CUP-NEN (n)**	**5** (15.15%)
**Metastic spread**	**NEN-(Mx) (n)**	**131 (100%)**
	hepatic	59 (45.0%)
	lymphnode	48 (36.6%)
	bone	19 (14.6%)
	pancreatic	5 (3.8%)
Ki-67 (%)*		6* (range, 1–17)
Cromogranin A* **(μg/l)**		632* (range, 94–12600)
**PRRT**	Number of cycles	3.3* (IQR, 3–4)
	Administered dose	200 mCi (7,4 GBq)

Detailed therapy and patient characteristics in systematic overview: *GEP-NEN*, gastro-enteropancreatic neuroendocrine neoplasm; *CUP-NEN*, neuroendocrine neoplasm of unknown primary

* = median and range/ *IQR*, interquartile range; *PRRT*, peptide receptor radionuclide therapy; *GBq*, gigabequerel, *mCi*, millicurie.

A total of 131 lesions (liver: n = 59, lymph nodes: n = 48, bone: n = 19, pancreas: n = 5) were analyzed. Of the 131 lesions, 104 lesions had been assigned to the RLs and 27 to the NRLs according to morphologic assessment [23]. There was no significant difference in the functional tumor volume (FTV) between both response groups prior treatment with [^177^Lu]DOTATATE-PRRT (p > 0.05).

### Lesion response

Assessment of the pre-therapeutic lesion diameters revealed 19.2 mm (IQR, 13.7–33.3 mm; range, 10.0–100.0 mm) for RL and 19.6 mm (IQR, 14.2–35.0 mm; range, 12.0–104.6 mm) for NRL. After 4-month follow up analyses of the RLs showed a significantly decreased median lesion size in RL of 16.9 mm (IQR, 13.0–29.5; range, 0.0–75.0; p < 0.001). Assessment of the NRLs demonstrated a significant increase in diameter to 23.2 mm (IQR, 16.1–40.0; range, 10.0–124.6; p < 0.001). Kruskal-Wallis test depicted a highly significant difference in the diameter of RLs and NRLs after two cycles, i.e. 4-months, of [^177^Lu]DOTATATE-PRRT (16.9 mm; IQR, 13.0–29.5 vs. 23.2 mm; IQR, 16.1–40.0; p < 0.001).

After 12 months follow-up, the median lesion size in RLs showed a reduction in diameter to 13.9 mm (IQR, 10.2–24.0; range, 0.0–63.0). The median lesion size in NRLs increased to 29.0 mm (IQR, 19.0–49.0; range, 15.0–136.4) after treatment. A highly significant difference was detected in the diameter of RLs and NRLs compared at initial measurement and after treatment (13.9 mm; IQR, 10.2–24.0 vs. 29.0 mm; IQR, 19.0–49.0; p < 0.001).

**Fig 1:**
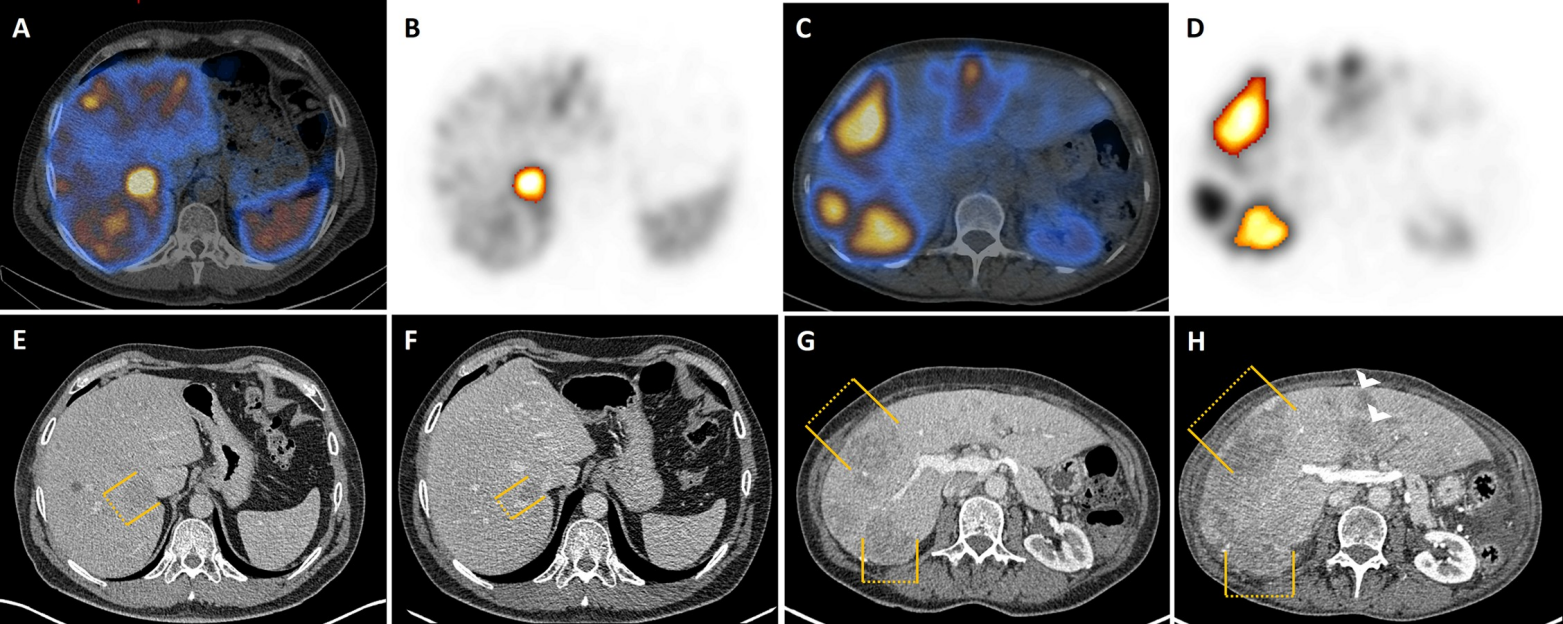
Representative responding lesion (RL) and non-responding lesion (NRL). Baseline SPECT/CT during dosimetry (24h p.i.). Representative example of one responding lesion in liver segment VI (A) of a 48-year-old man suffering from metastatic duodenal NEN. The lesion can be detected on SPECT (B) and is illustrated by orange bars on CT at baseline (E) and after 12 months follow-up (F). The lesion decreased from 3.9 cm with a functional SPECT-volume of 19.7 ml to 2.3 cm after 4 cycles’ [^177^Lu]DOTATATE-PRRT and 12 months follow-up, respectively. Images (C,D,G,H) showing a typical example of non-responding lesions. A 59-year-old woman suffering from pancreatic (P)-NEN with predominant liver metastases. Hepatic metastases can be detected on CT (G) as well as on SPECT (D) and image fusion, SPECT/CT (C). The lesions’ measurements are displayed by orange bars. Two exemplary lesions in liver segment VII and VIII (G) are shown with a maximum diameter of 3.5 cm (functional tumor volume [FTV] 26.4 ml) and 4.6 cm (FTV 34.0 ml) prior to treatment. After two cycles of [^177^Lu]DOTATATE-PRRT, tumor diameters increased to 4.2 cm (segment VII) and 7.0 cm (segment VIII). As an additional finding, new metastases are highlighted with white arrow heads (H).

Fig 1 presents a typical example of an RL, and a representative case of an NRL.

### Potential predictive relevance of ASP

**Fig 2:**
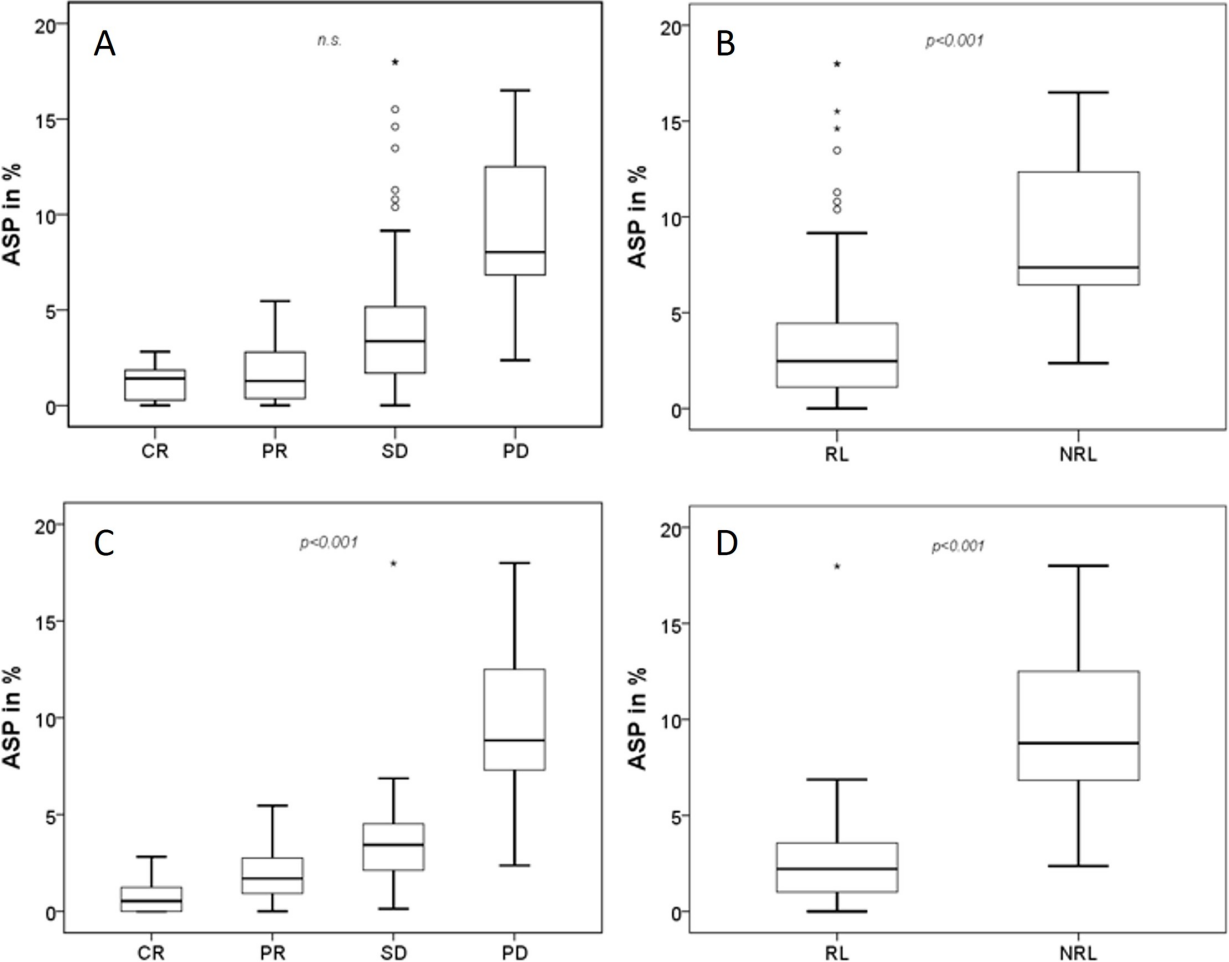
Box plot analysis of ASP. ASP clustered by RECIST criteria after (A) 4 and (C) 12-months follow-up. B and D shows ASP for responding (RL) and non-responding lesions (NRL) for the corresponding follow-up examinations at 4 and 12 months, respectively.

Lesions with high ASP_[Lu]_ showed a significantly poorer response to PRRT: PD, 11.3 (IQR: 8.5–15.5; range, 2.4–21.3); SD, 3.4 (IQR: 2.1–4.5; range, 0.2–18.0). PR, 1.7 (IQR: 0.9–2.8; range, 0.0–5.5). CR, 0.5 (IQR: 0.0–1.3; range, 0.0–2.8; Kruskal-Wallis, p < 0.001; Fig 2A and 2C).

Mann-Whitney U test analysis revealed a significantly lower ASP_[Lu]_ in RLs (2.9%; IQR, 0.9–3.9; range, 0.0–18.0) compared to NRLs (11.3%; IQR, 8.5–15.5; range, 2.4–21.3; p<0.5; Fig 2B and 2D)

**Fig 3:**
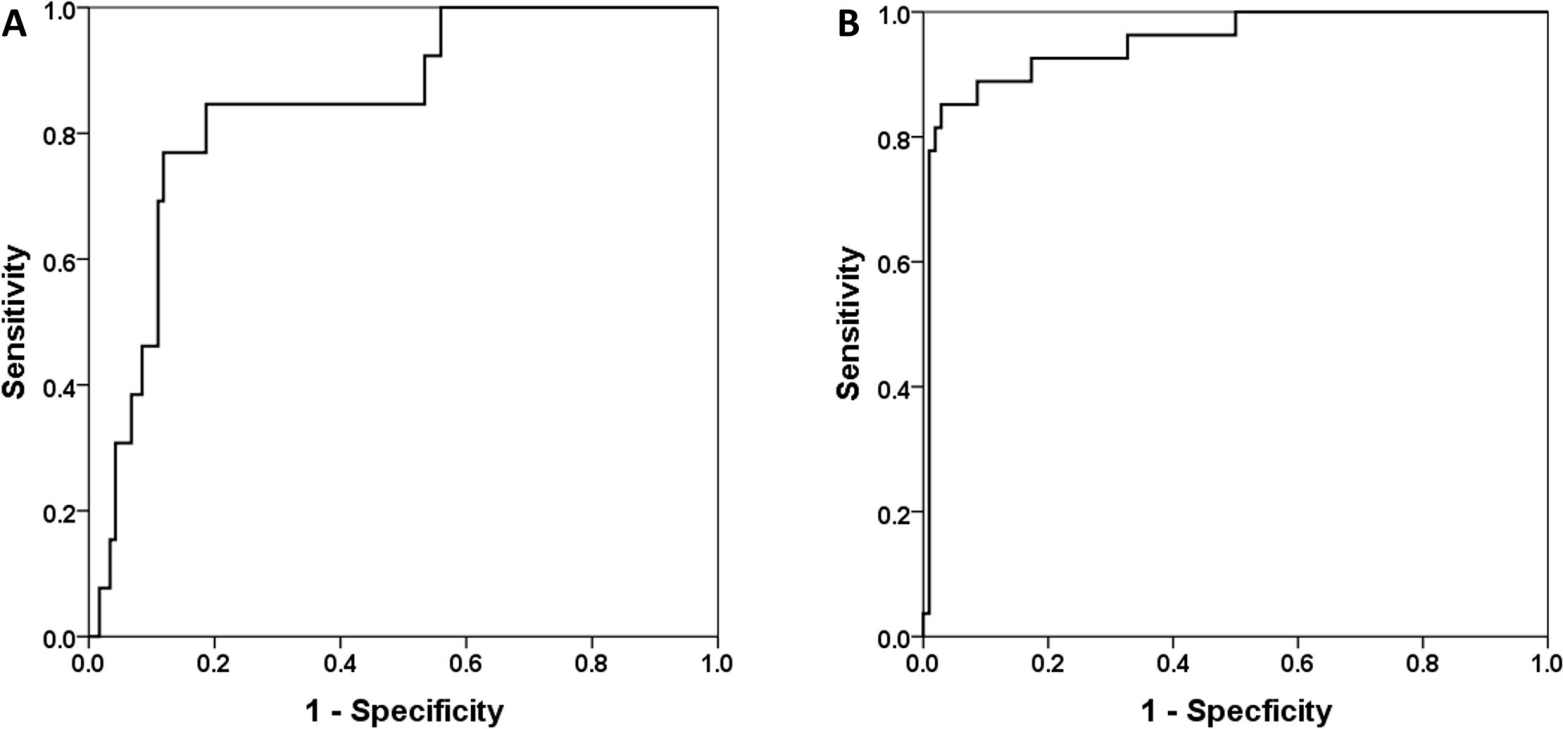
ROC curve comparison—4 and 12 months follow-up. ROC for the association of ASP with morphological treatment response (measures 4 months post first PRRT revealed an AUC = 0.85, p<0.001; A). The optimal cutoff for ASP in prediction of NRL was >6.39% (sensitivity and specificity, 77.0% and 88.0%). After 12 months (B), ASP showed an AUC of 0.94, p<0.001. The optimal cutoff for ASP was >5.45% (sensitivity and specificity, 96% and 82%).

Receiver Operating Characteristics (ROC) analysis revealed the highest area under the curve (AUC) for ASP to separate between RLs and NRLs after 4 months (AUC 0.85, p<0.001). The optimal cutoff value from ROC analysis was 6.39% (sensitivity and specificity, 77.0 and 88.0%; Fig 3). According to ROC-analysis, ASP after 12 months showed a highly significant AUC of 0.94 (p <0.001) to differentiate between the two response groups. The optimal cutoff for ASP was >5.45% (sensitivity and specificity, 96.0% and 82.0%, respectively; Fig 3).

### Correlation of ASP_[In]_ and ASP_[Lu]_

**Fig 4:**
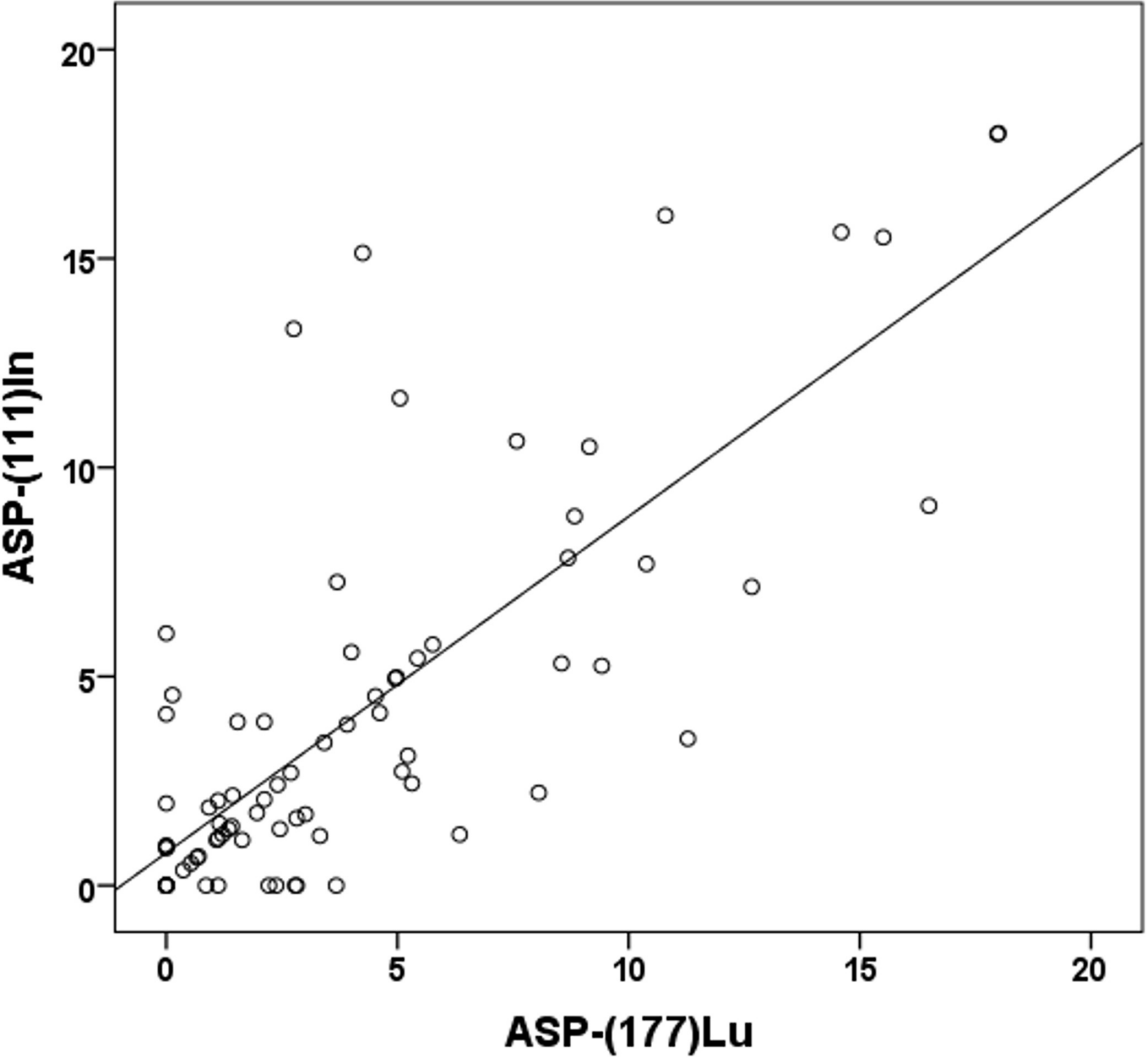
Spearman's rank correlation ASP_[In]_ and ASP_[Lu]_. Scatterplot showing the correlation of ASP estimated from pre-therapeutic [^111^In]-octreotide SPECT/CT [ASP-(111)In] and intra-therapeutic ASP [ASP-(177)Lu] (*ρ* = 0.72, p <0.01).

From the overall cohort of 33 patients, 20 patients, containing a pre-therapeutic [^111^In]octreotide SPECT/CT (77 lesions; liver: n = 60, lymph nodes: n = 11, bone: n = 4, pancreas: n = 2) were analyzed in a subgroup. The optimal threshold for the pre-PRRT estimated ASP_[In]_ was <5.12% (sensitivity 90% and specificity 93%) and for the intra-therapeutically estimated ASP_[Lu]_ <5.02% (sensitivity 92% and specificity 89%). The correlation coefficient was *ρ* = 0.72 (p <0.01, Fig 4). The mean absolute difference between ASP_[In]_ and ASP_[Lu]_ was -0.04% (95% Limits of Agreement, -6.1–6.0). 10/77 lesions (7/60 liver, 1/10 lymph nodes, 2/6 others) were discordant (absolut difference>5%).

## Discussion

In this retrospective study, we investigated the spatial heterogeneity (ASP) in SSR-positive/ functional metastatic GEP-NEN, using intra-therapeutic SPECT/CT data derived from the first cycle PRRT. Using this methodology we showed a strong association of ASP with morphological treatment response in patients undergoing PRRT with [177Lu]-DOTATATE. Even in diagnostic and in therapeutic imaging using [^111^In]-OctreoScan or [^177^Lu]-PRRT we demonstrated a strong correlation, resulting in high sensitivity and specificity.

A number of studies have already dealt with different texture analyses aimed at differentiation between benign and malignant lesions [25]. The tumor heterogeneity in lung carcinoma was evaluated by CT texture analysis. Considering a quite good contrast in lung tissue the quantification within lung tumors can be easily assessed. However, this is not the case within liver or other organ lesions. Here CT delination would be more challenging until semi-automatic segmentation of tumor delineation is limited due to worse tissue contrast in CT images. Lesions have to be evaluated in a manually, volumetric analysis–slice by slice. MRI delivers a better soft tissue contrast and the potential for accurate segmentation of metastases in the liver and other organs, respectively. Besides, several other studies worked with texture oriented analysis of morphologic data from CT or MRI in various entities (e.g. lung carcinoma [26] or colorectal cancer [27]), our analysis was based on SSR expression estimated by functional imaging. The predictive and prognostic importance of metabolic spatial heterogeneity of tumor lesions was also demonstrated for different cancer types e.g. using 18F-fluorodeoxyglucose (FDG)-PET/CT with partly promising results [22, 28]. Corresponding observations have also been published by Werner et al. who reported on the tumor entropy as a surrogate parameter assessed by [^68^Ga]DOTATATE- or [^68^Ga]DOTATOC-PET/CT in patients with late-stage GEP-NEN prior PRRT [16].

Heterogeneity was the favored parameter in distinction between responder and non-responder. In particular, heterogeneity parameters (such as homogeneity, correlation, size variation and short zone emphasis and “entropy” with the highest AUC of 0.70) outperformed conventional parameters like standardized uptake value (SUV) and total receptor expression. In line with these findings, heterogeneity analysis of thyroid cancer showed only significant results in a patient-based setting. Despite our results, in a lesion based analysis only the parameter “entropy” demonstrated with an AUC of 0.73 and a definitely lower sensitivity of 67% and a specificity of 75% response prediction [29]. For adequate therapy-monitoring lesion-based control is always preferable compared to patient-based follow-up. In NEN the mitotic count (MC) and Ki-67 are the most common and reliable biomarkers for cell-proliferation and thus, indicators for biological phenotypes [30, 31]. Although MC was used as the biomarker for GEP-NEN during the last decades, finally Khan et al. [32] showed the disparity of MC and Ki-67 and favored Ki-67 as a prognostic marker in grading. Actual Ki-67 plays the major role in dividing GEP-NEN into different grades of disease (G_1-3_ NEN/ G_3_-NEC) which directly affects therapy management [33]. Unfortunately, the Ki-67, as a prognostic marker, delivers some pitfalls, physicians have to be aware of. Assessment of Ki-67 depends on expertise of the reporting pathologist [34] and core biopsies represent only a small tumor sample, which impedes accurate heterogeneity assessment, especially of intermediate G_2_-lesion [35]. Lastly, Ki-67 index is known to fluctuate in some patients, not only with the choice of therapy but also during the time of treatment [36, 37]. Considering these findings, non-invasive and reliable whole-body assessment of inter-lesional heterogeneity may reflect the whole tumor burden in a more representative way–taking the divergent tumor areas into account. Even the well-established urine and serum-based parameters for treatment monitoring and follow-up, such as 5-hydroxyindole acetic acid (5-HIAA) [37] and Chromogranin A (CgA) [38], failed as prognostic biomarker. Since 18–33% of all patients with metastatic GEP-NEN show PD after PRRT, ASP might deliver a reliable surrogate parameter in selecting potential non-responder [39, 40]. Especially, those patients can benefit from an early therapy-switch. Applying the ASP in pretherapeutic octreotide-scintigraphy, we could recently demonstrate that low ASP predicts response to PRRT [13]. However, when dealing with elaborative predictive parameters, it is advantageous to use comparable DOTA-conjugates, such as intra-therapeutic [^177^Lu-DOTA^0^,Tyr^3^]octreotate, with equal SSR-affinity to ensure robustness of the test and to minimize varieties.

Nevertheless, subgroup analysis presented a high correlation of ASP_[In]_ and ASP_[Lu]_ and thus, validated the method. The marginal better area under the curve for ASP, obtained in pretherapeutic SPECT/CT imaging, might has been affected by a smaller study population bias.

More recently, functional imaging of NEN with ^68^Ga-labelled DOTA analogues (e.g., TATE/ TOC/ NOC) is the preferred SSR-imaging in follow-up examinations, due to better sensitivity, spatial resolution and less radiation [41]. Although there is still limited data available regarding PET-based follow-up, the ENETS consensus guidelines recommend MRI/ CT every 3–12 months and a receptor imaging in G_1-2_ P-NEN each 12–24 months after treatment. If there is progression suspected, even anytime earlier [42]. Moreover, the post-therapeutic scan is performed during therapeutic procedure (e.g. for dosimetry) immediately after treatment (24 h p.i.). Providing an early surrogate for tumor response from analyzing. Our results suggest that ASP may deliver a strong approach in intra-therapeutic risk monitoring, indicating that follow-up intervals, e.g. CT every 3–12 months, [^68^Ga]DOTANOC-PET/CT every 12–24 months, can be even prolonged. An encouraging aspect is the synergistic character of the ASP evaluation. ASP analysis uses functional data from standard diagnostics in patients with GEP-NEN or from dosimetry (in treatment validation) providing benefits without an additional test.

Different limitations may arise: Contrary to other qualitative visual scores, the ASP may deliver a semi-automatic parameter, which ensures reproducibility and robustness in prediction response. Nevertheless, the ASP is not a scanner independent surrogate parameter. It can be hypothesized that ASP is in small lesion affected by partial volume effect. Mainly due to remarkable progress in functional imaging and resolution, lesions smaller than 2.5ml [22], which used to appear with symmetrical sphere shape, can be depicted these days in less than a few millimeters in size. Thus, modern SPECT-devices, such as cadmium-zinc-telluride (CZT) detectors, exhibit a higher spatial resolution [43]. This might avoid adulteration of the ASP in smaller lesions. The limitations of our study are the small sample size, no comparison group and retrospective design. Nevertheless, considering GEP-NEN are commonly rare tumor entity [1], we were able to provide valuable findings and demonstrate significant effects that show some support for our hypotheses. However, further validation of ASP as a potential predictive marker is still required.

## Conclusion

Pre- and intra-therapeutic ASP shows a strong correlation with morphological treatment response and might represent a potential predictive marker for PRRT. Further assessment of its predictive value is worthwhile and might improve therapeutic decision making in NEN.
